# Enhancing the Mechanical Performance of Bleached Hemp Fibers Reinforced Polyamide 6 Composites: A Competitive Alternative to Commodity Composites

**DOI:** 10.3390/polym12051041

**Published:** 2020-05-02

**Authors:** Francisco J. Alonso-Montemayor, Quim Tarrés, Helena Oliver-Ortega, F. Xavier Espinach, Rosa Idalia Narro-Céspedes, Adali O. Castañeda-Facio, Marc Delgado-Aguilar

**Affiliations:** 1 Facultad de Ciencias Químicas, Universidad Autónoma de Coahuila. Blvd. V, Carranza and Eng. José Cárdenas s/n, Col. República, C.P, 25280 Saltillo, Mexico; franciscoalonso@uadec.edu.mx (F.J.A.-M.); rinarro@uadec.edu.mx (R.I.N.-C.); adali.castaneda@uadec.edu.mx (A.O.C.-F.); 2 Department of Chemical Engineering, LEPAMAP Group, University of Girona, 17003 Girona, Spain; helena.oliver@udg.edu; 3 Department of Organization, Design, Development and Product Innovation, Business, University of Girona, 17003 Girona, Spain; francisco.espinach@udg.edu

**Keywords:** biocomposites, cellulose, mechanical properties, micromechanics

## Abstract

Automotive and industrial design companies have profusely used commodity materials like glass fiber-reinforced polypropylene. These materials show advantageous ratios between cost and mechanical properties, but poor environmental yields. Natural fibers have been tested as replacements of glass fibers, obtaining noticeable tensile strengths, but being unable to reach the strength of glass fiber-reinforced composites. In this paper, polyamide 6 is proposed as a matrix for cellulosic fiber-based composites. A variety of fibers were tensile tested, in order to evaluate the creation of a strong interphase. The results show that, with a bleached hardwood fiber-reinforced polyamide 6 composite, it is possible to obtain tensile strengths higher than glass-fiber-reinforced polyolefin. The obtained composites show the existence of a strong interphase, allowing us to take advantage of the strengthening capabilities of such cellulosic reinforcements. These materials show advantageous mechanical properties, while being recyclable and partially renewable.

## 1. Introduction

Short fiber-reinforced polymer composites are formulated and designed with the intention of obtaining new materials with enhanced properties with respect to the matrix [1]. These properties can vary, but usually are related with the mechanical, economical or environmental performance of the composites [2]. Using a reinforcement that is cheaper than the matrix can deliver economic competitiveness. In some cases, the reinforcements will increase the mechanical properties, and the composite will show better strength and stiffness than the matrix, but this depends on the interactions between the matrix and the reinforcement in the interphase [1,3]. Therefore, materials with weak interphases, even if they achieve an economic advantage, will decrease the strength of the matrix. Thus, in order to obtain economic and mechanical advantages, the reinforcement must be cheap and must ensure the creation of a strong interphase with the matrix. On the other hand, to increase the environmental advantages, the reinforcement must be renewable, locally available and recyclable or biodegradable [4].

Cellulosic fiber-reinforced composites are materials that aspire to achieve mechanical, economic and environmental advantages. Natural fibers can be obtained from a large number of sources, from annual plant to agroforestry waste, ensuring competitive cost, as some have negligible values, availability, renewability and biodegradability. Thus, natural fibers seem to be the best option as polymer reinforcement. Nonetheless, natural fibers have some drawbacks. On one hand, its hydrophilic nature hinders the interactions with hydrophobic matrices, and obtaining strong interphases is not possible without fiber treatments or the use of coupling agents [5,6,7]. On the other hand, natural fibers cannot be exposed to temperatures above 200 °C because, otherwise, cellulose degrades quickly [1].

Polyolefins have been the matrices used more often to prepare natural fiber-reinforced composites [8,9,10,11]. On one hand, polyolefin like polypropylene (PP) or polyethylene (PE) are well known materials with notable mechanical properties and competitive prices. Most of the polyolefins can be processed under 200 °C, ensuring low degradation of the cellulose during composite preparation. Furthermore, the compatibilization between cellulosic fibers and polyolefin has been profusely studied in literature [12,13,14]. This literature shows how fiber treatments that decrease the percentage of lignin in the surface and the use of coupling agents like maleic acid aid in obtaining composites with notable mechanical properties [1,15,16]. Nonetheless, the competitiveness of such materials must be compared to already available commercial ones. In this sense, glass fiber (GF)-reinforced polyolefins are industrial commodities. These materials were designed with the intention of overcoming the mechanical properties of polyolefin and have been profusely used by automotive and industrial design companies [17]. Notwithstanding, glass fiber-based materials have some drawbacks, mainly, its composites are not recyclable, the fibers wear out the equipment by friction, and are noticeably heavier than the matrices. Literature shows that, with natural fiber-reinforced polyolefins, it is possible to obtain strength and stiffness similar to sized GF-reinforced materials. Nonetheless, GF-reinforced polyolefins deliver strength and stiffness that cannot be obtained with natural fiber-reinforced composites [1,18,19]. 

Knowing the main aspects that affect the mechanical properties of a composite is important in order to achieve the mechanical properties of a glass fiber (GF)-reinforced polyolefin composite. In the case of mold injected short fiber-reinforced materials, such aspects are: the percentage of matrix and reinforcement, the nature of the phases, the morphology, mean orientation and dispersion of the reinforcements and the strength of the interphase [20,21]. While all the cited parameters are important and equalize the properties of a composite, it is impossible to increase the strength of the matrix without a strong interphase. Moreover, from a strength point of view, the feeblest component of a composite is usually the interphase. The strength of the interphase depends on the different interactions between the fibers and the matrix, and is feebler than the matrix. Von Mises criteria can be used to obtain an upper bound for the strength of the interphase, comprising the ratio between the tensile strength of the matrix and the square root of three [22]. Thus, the strength of the interphase is limited by the strength of the matrix. Hence, it is only possible to fully deploy all strengthening capabilities of the reinforcement if a strong interphase is obtained. In any case, the intrinsic tensile strength of a cellulosic fiber is lower than GF. This can be overcome by increasing the percentage of reinforcement in the composite, but such increases decrease the mold flow index (MFI). Furthermore, the reduction on MFI complicates a correct dispersion of the fibers. Therefore, the use of another matrix with higher tensile strength, which aids in obtaining strong interphases, can be a solution.

Polyamide 6 has received some attention as a possible matrix for short fiber-reinforced composites [23,24,25,26]. Nonetheless, to the best knowledge of the authors, only a few use cellulosic fibers as reinforcement [27,28,29,30]. Moreover, the composite materials reinforced with cellulosic fibers show low increases of its tensile strength against reinforcement content and thus a feeble interphase [30]. Polyamide 6 has been successfully reinforced with glass fibers, showing the ability of such matrix to create strong interphases. Literature shows that a polyamide 6 (PA6) with a tensile strength of 51.8 MPa can reach up to 96.2 MPa when it is reinforced with a 30 wt % of GF. The main drawback for PA6 as cellulosic fibers matrix is its processing temperature of 245 °C, but minimizing the exposure of the cellulosic fibers to such temperatures can decrease its degradation.

Glass fiber-reinforced PP composites at 30 wt % reinforcement contents show tensile strengths of 58.5 and 79.6 MPa for the sized glass fiber and GF with malleated polypropylene as coupling agent (MAPP), respectively [31]. These tensile strengths are near the tensile strength of a neat PA6 matrix (69.7 MPa). Thus, it is plausible to think that PA6 based composites can reach such strength if a minimally strong interphase between cellulosic fibers and PA6 is obtained.

In this paper, PA6 based composites at 20 wt % content of different cellulosic reinforcements were formulated, prepared and tensile tested. The presence of cellulosic fibers increased the tensile strength of the matrix. Nonetheless, the results showed the impact of the fiber treatments on the tensile strength of the composites. The best results were obtained with bleached hemp and flax fibers, with a slight advantage for hemp fibers. In order to increase the economic advantages of the composites, hemp was chosen instead of flax, as it is cheaper. Composites with hemp contents ranging from 10 to 30 wt % were prepared and tensile tested. The results showed a linear evolution of the tensile strength against reinforcement contents. A micromechanical analysis was made, in order to evaluate the strength of the interphase. The results showed the existence of interactions between PA6 and the cellulosic fibers, and the creation of an interphase. The obtained composites showed higher tensile strengths than GF-reinforced PP composites. The studied composites showed mechanical properties, and environmental and supposedly economic advantages over commodity materials.

## 2. Materials and Methods

**Figure 1:**
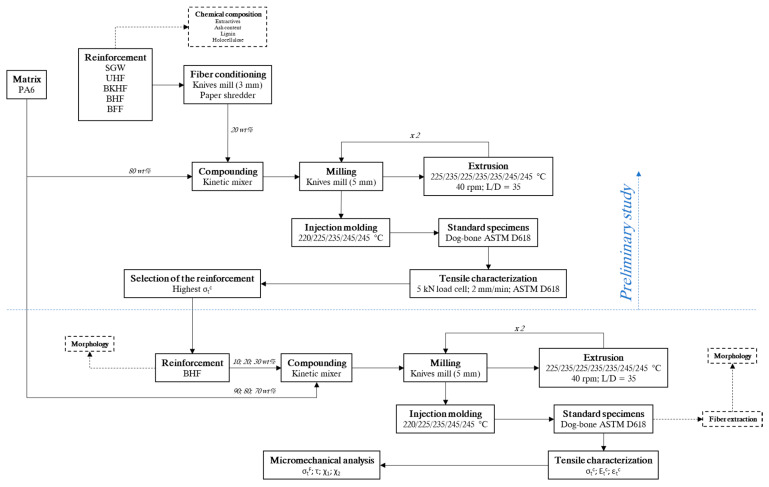
Workflow of the present study.

Figure 1 shows the workflow for the conducted research, from the raw materials to the evaluation of the obtained composites.

### 2.1. Materials

TECHNYL C—206 F Natural R polyamide 6 (PA6) was selected as polymer matrix and was provided by the Resinex Spain, S.L. (Vilallonga del Camp, Spain). As the reinforcement, different fibers were used such as: stone groundwood pulp (SGW), untreated hemp filaments (UHF), bleached kraft hardwood fibers (BKHF), bleached hemp fibers (BHF) and bleached flax fibers (BFF). SGW was kindly provided by Zubialde S.A. (Aizarnazábal, Spain), UHF by Cal Sagristà (Solivella, Spain), BKHF by Torraspapel, S.A. (Zaragoza, Spain) and both BHF and BFF by Celulosa de Levante S.A. (Tortosa, Spain). All the reagents used for fiber extraction and chemical characterization were provided by Sharlab, S.L. and were used as received with no further purification.

### 2.2. Methods

#### 2.2.1. Chemical and Morphological Characterization of the Fibers

Chemical composition of the fibers was determined according to different TAPPI standards. Extractives were determined in accordance to TAPPI T204 cm-97. Ash content was determined according to TAPPI standard T211. Acid-insoluble lignin, also known as Klason lignin, of the extractive-free samples was determined according to TAPPI standard T222. Cellulose and hemicellulose content was determined by high performance anion exchange chromatography (HPAEC), similar methodology was previously described by [32,33]. Morphological analysis was carried out in a MorFi Compact analyzer (Techpap, France) as described elsewhere [1,22,34].

#### 2.2.2. Composites Preparation and Injection Molding of the Standard Specimens

Depending on the original state of the reinforcement, the selected raw materials were processed by means of different methods. In the case of SGW and UHF, which were provided as a dried pulp and long filaments, respectively, they were passed separately through a knives mill provided with a 3 mm metallic mesh at the bottom. On the contrary, BKHF, BHF and BFF were provided as pulp laminates and were passed through a conventional paper shredder. All the reinforcements were dried at 60 °C until constant weight before being incorporated to the polymer matrix.

Composite materials at 10% to 30% (wt/wt) of reinforcement were prepared in a Gelimat kinetic mixer (Mod. G5S, Ramsey, New Jersey, USA) as described by [1]. Due to the friction generated between fibers, PA6 pellets and the sides of the drum, the blend temperature rose to 230 °C after 3 min. At this temperature, the blend was discharged. To enhance fiber dispersion, composites were extruded twice in a single-screw extruder Eurotecno 3035 D (L/D = 35) with the temperature profile set at 225/235/225/235/235/245/245 °C. The speed of the screw was set at 40 rpm. Finally, the extruded materials were granulated again using the abovementioned knives mill (5 mm mesh) and kept in an oven at 80 °C. 

Standard specimens were obtained by means of injection molding, using a 220 M 350-90U injection molding machine (Arburg, Germany). The processing temperatures of the five heating zones were 220/225/235/245/245 °C, corresponding the last to the injection nozzle. The working pressure was increased in accordance with higher fiber content, ranging from 500 to 900 bar. The obtained specimens were conditioned at 23 °C and 50% of relative humidity for 48 h, as required by ASTM D618, before testing.

#### 2.2.3. Characterization of the Composites

Tensile tests were conducted as described in literature [21,35]. The rheological behavior of the fiber-reinforced PA6 composites was studied by means of a melt flow index (MFI) tester (CEAST, Melt Flow Quick Index). Tests were performed in accordance with ISO standard 1133 at 240 °C and a weight of 3.8 kg. Scanning electron microscopy (SEM) of the fracture zones was performed. For this, the fracture surfaces were gold sputtered and images were taken with a Zeiss Ibera DSM 960A microscope (Madrid, Spain). 

#### 2.2.4. Extraction of the Fibers from the Composites

The PA6 matrix was dissolved in a Soxhlet apparatus. The extraction was lasted for 72 h by means of reflowing glacial acetic acid. Then, the extracted fibers were washed with acetone and water. Finally, the residual solvents were eliminated by means of drying the recovered fibers at 105 °C for 24 h.

#### 2.2.5. Tensile Testing Modelling

The micromechanics of the tensile strength was studied by using different models, based on a modified rule of mixtures (Equation (1)) [36,37]:

(1) $$\sigma_{t}^{c}=f_{c}V^{f}\sigma_{t}^{f}+\left(1-V^{f}\right)\sigma_{t}^{m*}$$

where $\sigma_{t}^{c}$ is the tensile strength of the composite, $f_{c}$ is the efficiency factor, $V^{f}$ is the volume fraction of fiber in the composite, $\sigma_{t}^{f}$ is the intrinsic tensile strength of the fiber and $\sigma_{t}^{m*}$ is the tensile strength of the matrix at the strain at break of the composite. The $f_{c}$ can be decomposed into the orientation factor ($\chi_{1}$) and the length and fiber-matrix interphase factor ($\chi_{2}$).

However, the values of $\chi_{1}$, $\chi_{2}$ and $\sigma_{t}^{f}$ cannot be directly calculated or determined, as has been previously reported [38]. Thus, a Kelly and Tyson modified equation was proposed to obtain these composite micromechanic parameters [39], which was solved using the method proposed by Bowyer and Bader [40]. The Kelly and Tyson model is obtained in Equation (2).

(2) $$\sigma_{t}^{c}=\chi_{1}\left(\sum_{i}\left[\frac{\tau \cdot l_{i}^{f}\cdot V_{i}^{f}}{d^{f}}\right]+\sum_{j}\left[\sigma_{t}^{f}\cdot V_{j}^{f}\cdot \left(1-\frac{\sigma_{t}^{f}\cdot d^{f}}{4\cdot \tau \cdot l_{j}^{f}}\right)\right]\right)+\left(1-V^{f}\right)\sigma_{t}^{m*}$$

where *τ* is the interfacial shear strength (IFSS), $d^{f}$ is the mean diameter of the fiber and $l_{i}^{f}$ and $l_{j}^{f}$ are the mean lengths of the fibers classified as subcritical and supercritical, respectively. Bowyer and Bader proposed a numerical solution for the equation. This equation needs a value for the intrinsic Young’s modulus ($E_{t}^{f}$) that cannot be empirically calculated, so the application of the Hirsch model (Equation (3)) is required. The Hirsch model is, in fact, another approach based on the combination of parallel and series models of the rule of mixtures.

(3) $$E_{t}^{c}=\beta \left(E_{t}^{f}V^{f}+E_{t}^{m}\left(1-V^{f}\right)\right)+\left(1-\beta \right)\frac{E_{t}^{f}E_{t}^{m}}{E_{t}^{f}V^{f}+E_{t}^{m}(1-V^{f})}$$

where *β* is a parameter related to the stress transfer between fiber and matrix and is mainly affected by the fiber orientation, length and stress amplification effect at fiber ends [41]. As reported elsewhere, theoretical and experimental values can fit when 0.4 is assigned to *β* parameter [42]. $E_{t}^{m}$ is the Young’s modulus of the neat matrix.

## 3. Results and Discussion

### 3.1. Preliminary Study: Selection of the Reinforcement

**Figure 2:**
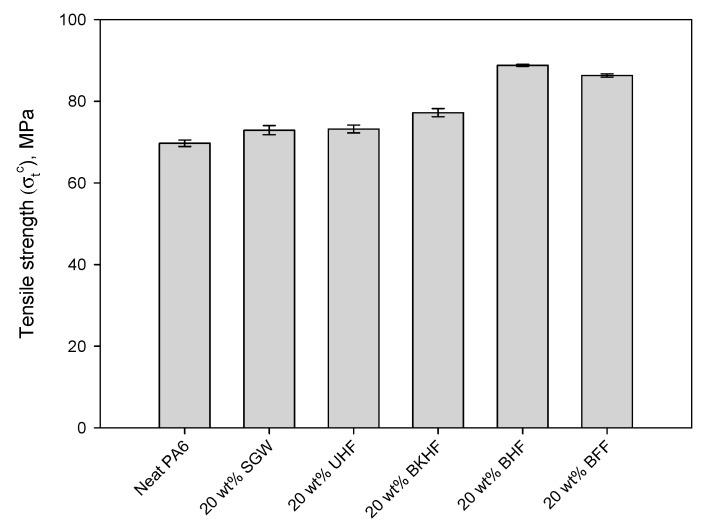
Tensile strength of neat polyamide 6 (PA6) and PA6-composites containing 20 wt % of stone groundwood pulp (SGW), untreated hemp filaments (UHF), bleached kraft hardwood fibers (BKHF), bleached hemp fibers (BHF) and bleached flax fibers (BFF).

As described in the previous section, different fibers were used as reinforcements and were characterized. For this, composites with a 20 wt % of SGW, UHF, BKHF, BHF and BFF were prepared using PA6 as the matrix and tested at tensile (Figure 2). In all cases, the tensile strength of the composite was higher than the neat matrix, which exhibited a tensile strength of 69.7 MPa.

**Figure 3:**
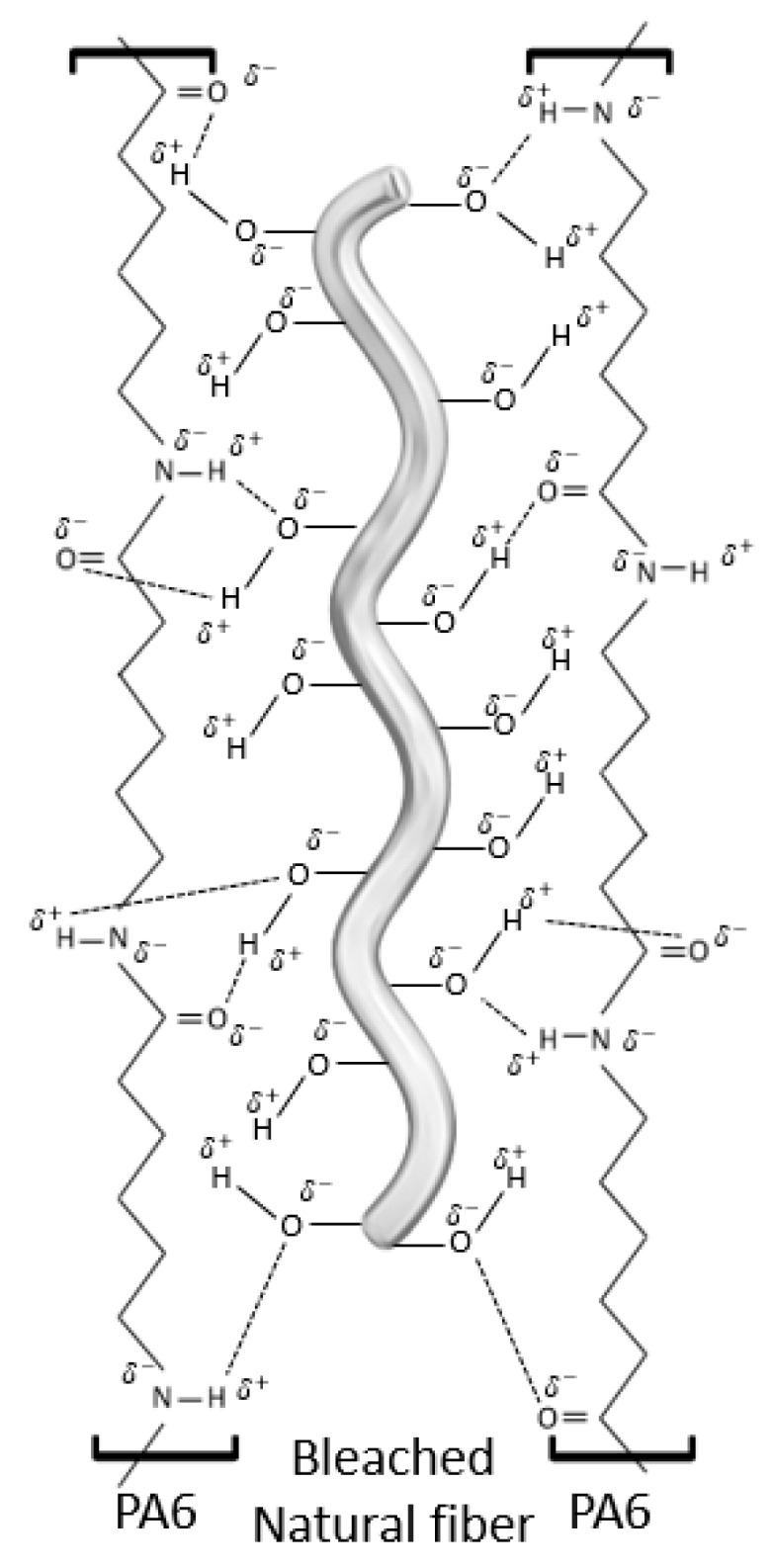
Proposed mechanism of bleached fibers anchoring onto PA6 surface.

As can be observed, the incorporation of 20 wt % of reinforcement enhanced the tensile strength of PA6 by 4.59%, 5.02%, 10.76%, 27.40% and 23.82% when SGW, UHF, BKHF, BHF and BFF were used as the reinforcement, respectively. It has been widely reported that the main factors influencing the tensile strength of composites reinforced with short and semi-aligned fibers are (i) the volume fraction of the reinforcement, (ii) the dispersion of the reinforcement within the matrix, (iii) the aspect ratio of the reinforcement, (iv) the intrinsic tensile strength of the reinforcement, (v) the orientation factor of the reinforcement, (vi) the interaction between fiber and matrix (IFSS) and (vii) the tensile strength of the matrix [1]. From these factors, there are some that can be assumed to be equal among the different composites and, thus, do not provide significant differences on the resulting mechanical properties of the composites. This is the case of the orientation factor, the tensile strength of the matrix and the volume fraction of the reinforcement. In fact, all of the composites were processed following the same methodology, the matrix was common for all the composites and the volume fraction was the same. In fact, in terms of orientation, the mean orientation angle is mainly obtained by the geometry of the injection mold and the equipment used to obtain the specimens. From the obtained results, and considering the differences between the fibers, it becomes apparent that the interaction between fiber and matrix plays a key role on the mechanical performance of the composites. Indeed, only those lignin-free fibers exhibited reinforcing capacities above 20%. Notably, the presence of lignin hindered the interaction between PA6 and the reinforcement, leading to presumably low IFSS. In fact, SGW and UHF exhibited a lignin content of 26.3% and 6.1% respectively, while the rest of the fibers only contained residual traces. Considering the structure of PA6 and that bleached fibers have mainly hydroxyl groups at their surface, the interaction between fiber and reinforcement is presumably based on hydrogen bonding and ionic interactions, as it is proposed in Figure 3.

However, the absence of lignin, extractives and ashes leads to fibers mainly composed of holocellulose (almost 100%) which, at the same time, includes both hemicellulose (8.51%) and cellulose (91.59%). It is well known that both hemp and flax exhibit higher cellulose content than those pulps coming from wood sources, such is the case of BKHF [21,43]. In fact, this higher cellulose content may lead to superior intrinsic mechanical properties in the case of the abovementioned annual plants than in the woody pulp. In addition to this, as reported elsewhere, the incorporation of bleached kraft pulps usually requires the use of dispersants such as diglyme to promote fiber dispersion within the matrix [4].

Thus, from the reinforcements above, BHF was selected to proceed with the study, mainly due to its higher reinforcing potential of PA6 and, in addition, the lower purchasing cost of hemp against flax.

### 3.2. Rheological and Tensile Characteristics of BHF-Reinforced PA6 Composites

The melt flow index (MFI) is an indicator of the rheological properties of composites. In fact, a decrease on the MFI of a material implies an increase in its viscosity, especially on its consistency factor from the Power-Law model. This viscosity, or MFI, is measured at a certain temperature under a certain load. MFI is usually measured to determine the processing parameters such as temperature, pressure and cooling time during injection molding.

As detailed above, BHF was selected as the potential reinforcement for PA6 natural fiber-reinforced composites. The fiber contents were set at 10 wt %, 20 wt % and 30 wt % and, after compounding, the MFI was measured. PA6 exhibited MFI values of 36.48 g/10 min, a value that was significantly decreased as the amount of BHF increased in the composites. In fact, the MFI accounted for 10.61, 5.75 and 1.92 g/10 min for the composites reinforced with 10 wt %, 20 wt % and 30 wt % of BHF, respectively. These MFI represent a decrease of this property of 70.92%, 84.24% and 94.74% for the abovementioned fiber contents. This drastic increase of the viscosity is mainly due to the presence of fibers that do not melt during their processing which, at the same time, hinder the free movement of PA6 chains [44]. In fact, similar results have been reported for other lignocellulosic-reinforced PA6 composites [45,46,47]. Considering that the test was conducted at 240 °C and that the injection pressure achieved a value of 900 bar when 30 wt %-reinforced composites were processed, it becomes apparent that for processing higher fiber content composites, the temperature needs to be increased. However, it is well known that pure cellulose degrades around 300 °C in inert atmosphere and this temperature is significantly lower in the presence of oxygen and under the shear stress at which fibers are exposed during processing. Thus, the low MFI of the 30 wt %-reinforced composites indicate that this is the maximum fiber content that can be incorporated and successfully processed into PA6 polymer matrix.

**Figure 4:**
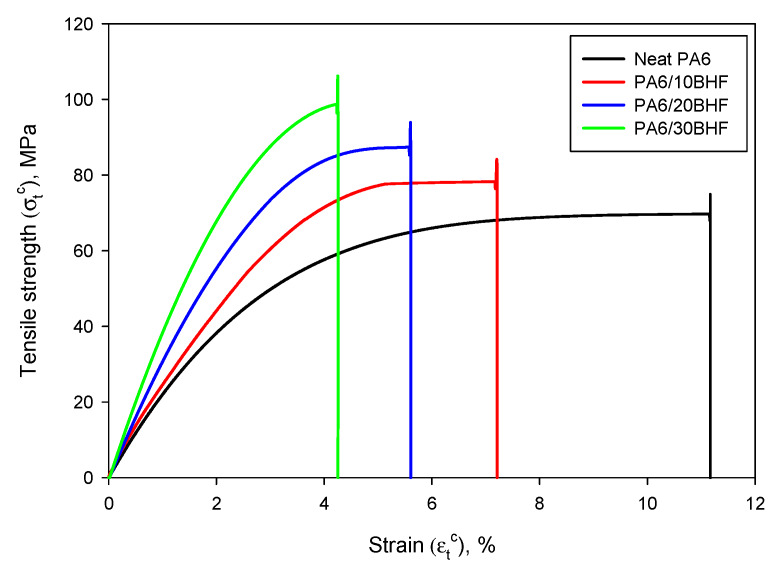
Stress-strain curves of neat PA6 and the BHF-reinforced composites.

The neat PA6 matrix exhibited a tensile strength of 69.70 MPa and was significantly improved by means of incorporating BHF. Concretely, the tensile strength was enhanced in a 12.27%, 27.36% and 41.21% for 10 wt %, 20 wt % and 30 wt % of BHF content in the composites. The tensile strength of the matrix at the breaking point of the composite ($\sigma_{t}^{m*}$) was obtained from experimental data and can be easily deducted from Figure 4, where the stress–strain curves can be observed.

As expected, $\sigma_{t}^{m*}$ proportionally decreased as fiber content increased, mainly due to the reduction of the strain caused by the reinforcement, thus, limiting the contribution of the matrix [48]. In addition, Figure 4 also shows that as the amount of BHF was increased, the strain at break was decreased, leading to clearly stiffer materials. Table 1 shows the tensile strength of the composites, the strain at break, the volume fraction and the Young’s modulus of the tested materials.

The tensile strength of the composites increased linearly with the volume fraction of BHF (R^2^ = 0.9991). In fact, this linear behavior indicates a good adhesion between the matrix and reinforcement at the interphase, as well as an efficient dispersion of the fibers inside the matrix. This adequate dispersion is also corroborated by the linear increase of the Young modulus [42,43]. Other researchers have already obtained similar results of tensile strength for natural fiber-reinforced PA6 composites. The highest values correspond to those reinforced with alkaline treated curauá (Ananas lucidus) fibers with reported tensile strengths of 87.00 and 90.20 MPa for 20 wt % and 30 wt % reinforcement, respectively [49]. Comparatively, the proposed composites in this work at the same reinforcement content exhibited higher mechanical properties than those reinforced with curauá fibers. One of the composites already in the market that can be considered as a commodity is glass fiber (GF)-reinforced polypropylene (PP). The GF content is usually around 30 wt % and it provides good mechanical properties. As an example, Kuram (2019) reported a tensile strength of 90.44 MPa when 30 wt % of GF was incorporated into PP [50]. Other authors have reported the effect of incorporating a 40 wt % of GF in presence of 6 wt % of coupling agent (maleated polypropylene, MAPP), achieving a tensile strength slightly higher than in the case of 30 wt %-reinforced BHF PA6 composites (101.2 MPa). Overall, the obtained results, at least in terms of tensile strength, bring to the light the feasibility of replacing such GF-reinforced PP composites by natural fiber composites. This could be advantageous not only in terms of environmental impact, but also for improving safety in production processes and reducing equipment attrition [1,51,52].

The strain at break ($\varepsilon_{t}^{C}$) was decreased as the amount of BHF was increased in the composites. The presence of fibers constrains the movement of PA6 chains, limiting the elongation that the resulting material can reach [30,53]. This effect has been previously observed for natural fiber-reinforced composites, as well as for those reinforced with mineral fibers [31]. Again, compared to GF-reinforced PP composites, it was found that the 30 wt % BHF-reinforced PA6 composite exhibited a similar $\varepsilon_{t}^{C}$ than the 40 wt% GF-reinforced PP composite [54]. This lower strain at break mainly comes from the interfacial adhesion between both phases in the composites, as well as for the higher stiffness and strength that BHF fibers exhibit compared to neat PA6 [55]. In this sense, the evolution of the Young modulus is, again, completely understandable, since a stiffer material was gradually incorporated into the matrix.

### 3.3. Analysis of Fractured Surface

**Figure 5:**
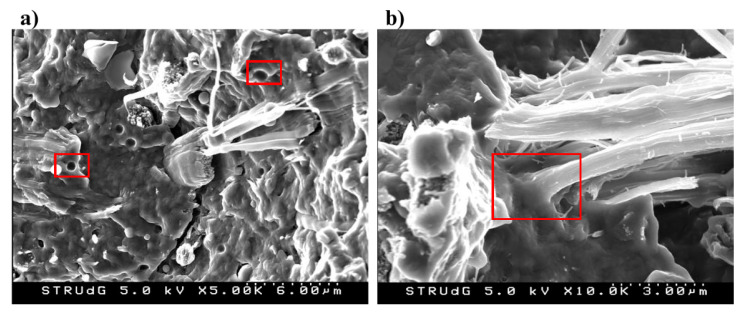
Scanning electron microscopy (SEM) images of the fracture surfaces of the 20 wt % BHF-reinforced PA6 composites. (**a**) voids in the structure of the composite PA6-Hemp fibers; (**b**) interface between fibers and matrix.

The interphase between BHF and PA6 was also studied by means of SEM of the fracture surfaces of the 20 wt %-reinforced composite (Figure 5). As shown in Figure 5a, a broken fiber can be observed, as well as a crack on the structure of the matrix. This indicates a strong interphase, which caused the failure of both phases during the fracture process. Moreover, Figure 5b reveals some other broken fibers and surface fibrillation, which also indicates the presence of a strong and good interphase between BHF and PA6 [56,57]. The formation of the PA6 interphase, thus, is proposed to be generated by means of two mechanisms. On one hand, mechanical anchoring, provided by the surface roughness of the fibers, which limits the fiber slippage within the matrix when submitted to mechanical stress. On the other hand, intermolecular forces by establishing hydrogen bonds between the hydroxyl groups of the BHF and the amide groups of the PA6. These mechanisms are detailed above in the proposed mechanism of interaction (Figure 3).

However, Figure 5a also reveals some voids in the structure of PA6 (left side). These voids may come from fiber slippage from the PA6 matrix. During compounding, although the mechanical tests reveal good dispersion, fiber agglomeration may occur, decreasing the effective surface area of the composite and, thus, limiting the fiber–matrix interaction. The higher polarity of PA6 compared to other thermoplastic materials, mainly due to the high presence of amide groups, make this thermoplastic material a good candidate to be used as polymer matrix for hydrophilic substrates with no need of using any coupling agent [2].

### 3.4. Evolution of the Fiber Morphology within the Composites

As detailed above, the aspect ratio of the reinforcement is a key parameter influencing the mechanical performance of short and semi-aligned fiber-reinforced composites. However, the fiber’s length is usually decreased during the processing of the composite due to the attrition phenomena. This effect is more pronounced in GF rather than in natural fiber, mainly due to the higher stiffness of GF compared to lignocellulosic fibers [15]. The diameter of the fibers usually decreases to a lesser extent or even remains constant, since shear forces have more of an effect on the longitudinal side of fibers rather than transversally. Table 2 shows the fiber diameter and length, as well as aspect ratio expressed as the ratio between the lengths weighted in weight and the diameters. The results are expressed as a function of the BHF content in the composite, as it has a direct effect on the change of morphology.

**Figure 6:**
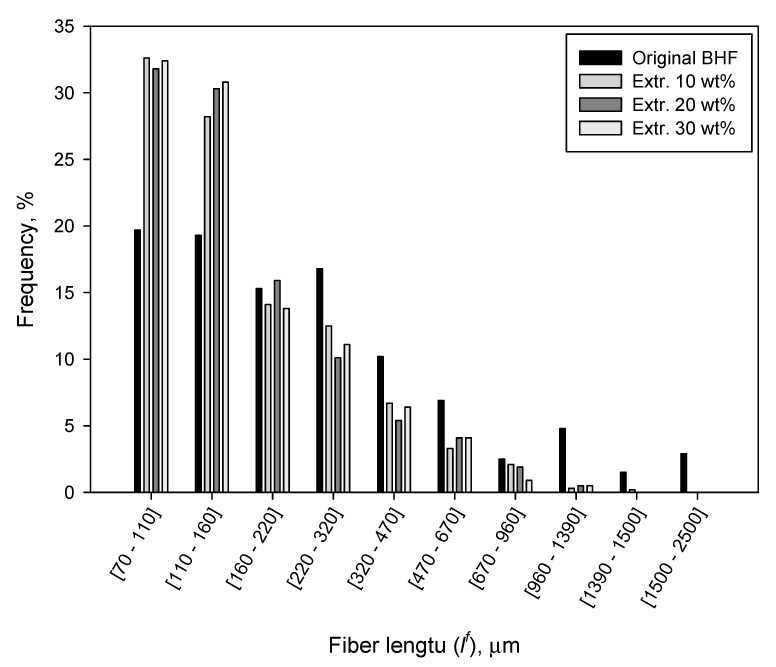
Length distribution of the original BHF and the BHF incorporated to PA6 at different content.

As it is possible to see, the length of the fiber was significantly decreased as a consequence of the extrusion and injection molding process. The evolution of lengths clearly showed the effects of shear forces experienced by the composite as the amount of fiber was increased. As previously observed by other authors, the diameter of the fibers remained almost constant from 10 wt % to 30 wt % BHF content, and close to the original value out of the composite (22.3 μm). As a consequence, the aspect ratio ($l_{ww}^{f}/d^{f}$) was significantly affected, experiencing a decrease of about 57% due to their incorporation into the composite [58,59]. The length decrease was also observed in the length distributions. As it is possible to see in Figure 6, the shape of the distribution significantly changed from the original BHF to those in the composite, apart from being shifted to left of the graph.

### 3.5. Micromechanical Analysis

As described above, the shape of the length distributions changed significantly from the raw fiber to those extracted from the composites. The distribution shifted to the left, with a noticeable increase of short fibers and a gradual disappearance of long fibers. This change of morphology has a direct effect on the mechanical properties of the composite, since the length of the reinforcements has a clear impact on the contribution of the fibers. Axial loads are transmitted to the fibers in different extent depending on their morphology [20].

Table 3 shows the experimental values used to solve the Kelly and Tyson modified equation using the solution provided by Bowyer and Bader. Strain levels were defined at 1/3 and 2/3 of the strain at break of the corresponding composite (Equation (2)). These points were chosen after reviewing the shape of the stress strain curve of the composites, with the objective of: increasing the distance between such points and avoiding the curved section of the curve, near the breaking point.

Table 4 shows the obtained results. The orientation factor gives information on the mean orientation of the fibers against the applied loads. Literature shows a high correlation between this mean orientation and the shape of the injection mold, the location of the gate and the parameters used during the injection [1]. The morphology of the fibers also has an impact on its orientation easiness [60]. Prior experiences showed that composite material mold injected with the same equipment than the used for the PA6-based composites returned orientation factors in the range of 0.25 to 0.35. Table 4 shows that the obtained orientation factors were inside the mentioned range. Thus, these results were considered valid. Literature shows a relation between the orientation factor and a mean orientation angle of the fibers (α) as: $\chi_{1}$ = cos^4^(α) [20]. Consequently, the mean orientation angles of the reinforcement were in the range of 42.3° to 43.4°. It must be noted that the orientation of the fibers inside a mold injected material is not regular, and three orientation zones can be identified: skin, shell and core. The mean orientation of the fibers changes noticeably with the zone [1,20]. Thus, the obtained mean orientation angle has only meaning for mechanical properties modelling purposes.

The interfacial shear strength change increased with the amount of reinforcement from 33.0 MPa to 40.0 MPa. This parameter gives notice to the strength of the interphase. These values are clearly superior to those obtained for other matrices like polypropylene, but it must be noted that the strength of the interphase is intimately linked to the strength of the matrix [20]. In this sense, Tresca (τ = $\sigma_{t}^{m}$⁄√3) and Von Mises (τ = $\sigma_{t}^{m}$⁄2) criteria place lower and upper bounds for the values of a strong interphase. Therefore, taking into account that the tensile strength of the PA6 was measured at 69.7 MPa, a strong interphase must return interfacial shear strengths in the range from 34.8 to 40.2 MPa. The obtained values are inside this range or near (in the case of the composite at 10 wt % of reinforcement). Hence, the presence of a strong interphase between the matrix and the reinforcements seems plausible. This implies the presence of chemical interactions between the fibers and the matrix, a good mechanical anchoring of the fibers, which is only possible if the matrix totally wets the reinforcements, and the absence of voids in the interphase [22]. 

The critical length of the fibers is determined by the outer area of the fibers and the ability to transmit the stresses from the matrix to the reinforcement (quality of the fiber–matrix interface). The critical length divides the reinforcements between subcritical (fibers with a length less than the critical length) and supercritical (fibers with a length greater than the critical length). The contribution of the reinforcement to the tensile strength of the composite changes noticeably from subcritical to supercritical fibers. Only supercritical fibers deploy all the strengthening capabilities of the reinforcement, by accumulating the loads and, finally, breaking. Subcritical fibers tend to pull out by interphase failure. Thus, the main contribution is expected to come from the supercritical reinforcements. The critical length decreased with the percentage of reinforcement, as a cause of the reduction of the mean length of the reinforcements (Table 2), but also as the increase of the interfacial shear strength. When an interphase is stronger, it is capable of transmitting more loads to the fibers, therefore, less length will be needed to reach higher loads inside the fiber.

The Kelly and Tyson equation was used to obtain the intrinsic tensile strength of the reinforcements. The values ranged from 1051 to 1178 MPa, with a mean value of 1110 MPa. This value situates the strength of bleached hardwood fibers at the level with natural reinforcements, like hemp or sisal [20,61]. Nonetheless, the intrinsic tensile strength of the cited fibers was obtained as polyolefin reinforcements, and some authors have discussed the relation between the chemical nature of the matrix and the yield of the strengthening capabilities of a reinforcement. Moreover, other researchers have commented on the expected differences between the properties of a fiber when are directly measured or obtained by micromechanics models [62]. Thus, the intrinsic tensile strength of BHF as PA6 reinforcement gives notice of its high strengthening capabilities. 

**Figure 7:**
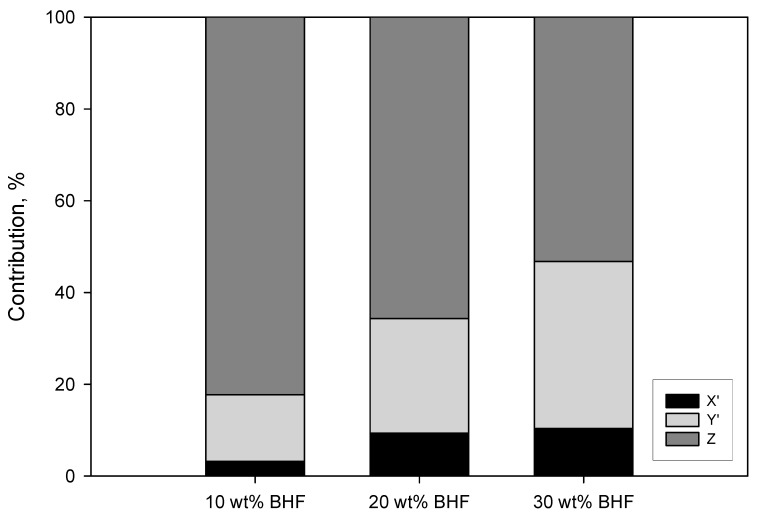
Percentage contributions of the phases of the composite to its tensile strength. Z is the matrix contribution to the tensile strength of composite, X’ is the subcritical fibers contribution and Y’ the supercritical fibers contribution.

Figure 7 shows the percentage contribution of the matrix (Z) and the subcritical (X’) and supercritical (Y’) fibers to the tensile strength of the composite.

The figure shows the noticeable contribution of the matrix to the tensile strength of the composites. PA6 contributes more than the 50% in all the cases. This can be expected from a matrix with such high tensile strength in comparison with polyolefin like polypropylene that release tensile strengths that are noticeably inferior. In spite of the high strengthening capabilities of the reinforcement and the strength of the interphase, the percentage differences between the tensile strength of BHF and PA6 decreased the percentage contributions of the fibers. Nonetheless, the contributions of the reinforcements are notable, and as expected, supercritical fibers made a higher contribution than subcritical. Taking into account the critical lengths (Table 4) and the length distributions (Figure 6), it can be observed that more than the 50% of the fibers are subcritical. Thus, a higher percentage of supercritical fibers can increase the contribution of the fibers noticeably and then the tensile strength of the composites. This can be fulfilled by increasing the strength of the interphase or by obtaining reinforcements with better aspect ratios. The obtained interfacial shear strengths limit the possibilities of obtaining stronger interphases. Therefore, the research of mixing and compounding methods that decrease or minimize the attrition phenomena and fiber shortening is an interesting field to obtain stronger PA6-based composites.

## 4. Conclusions

Polyamide 6 was reinforced with a series of cellulosic fibers. The results showed the influence of the chemical composition of the surface of the fibers on the tensile strength of the composites. The highest tensile strength was obtained with a bleached hardwood fiber-based reinforcement.

A study of the evolution of the tensile strength of BHF-based composites showed a linear evolution of such strength against reinforcement content. This is an indication of the existence of interactions between the matrix and the reinforcement, and thus, the existence of an interphase.

A micromechanics analysis was made in order to evaluate the strength of the interphase. The results showed that the reinforcements had mean orientation angles similar to polyolefin-based materials. The interfacial shear strength was in the range from 32 to 40 MPa, noticeably stronger than that exhibited by polyolefin-based composites. Moreover, these values are near Von Mises criteria, indicating the creation of a strong interphase. 

The contribution of the fibers was limited by its morphology. Due to the strength of the interphase, the critical length of the fibers increases noticeably and the number of critical fibers diminishes. Thus, researching composite preparation methods that allow for the minimization of fiber shortening due to attrition can aid us in obtaining even more strong materials. 

## Figures and Tables

**Table 1:** Tensile characteristics of the BHF-reinforced PA6 composites.

BHF (wt%)	V^f^ (-)	$\sigma_{t}^{C}$ (MPa)	$\sigma_{t}^{m*}$ (MPa)	$\varepsilon_{t}^{C}$ (%)	$E_{t}^{c}$ (GPa)
0	0	69.70 ± 0.86	69.70	11.38 ± 0.13	3.37 ± 0.21
10	0.077	78.25 ± 0.94	67.65	7.21 ± 0.12	4.33 ± 0.09
20	0.158	88.77 ± 0.46	64.87	5.77 ± 0.37	5.36 ± 0.11
30	0.244	98.42 ± 1.27	59.29	4.45 ± 0.26	6.69 ± 0.23

**Table 2:** Morphology of BHF prior to be incorporated to PA6 and after being incorporated at different reinforcement contents.

BHF (wt %)	V^f^ (-)	$l^{f}$ (µm)	$l_{w}^{f}$ (µm)	$l_{ww}^{f}$ (µm)	$d^{f}$ (µm)	$l_{ww}^{f}/d^{f}$ (-)
0	0	311.56	748.32	1336.1	22.3	59.91
10	0.077	196.76	332.52	563.5	21.7	25.97
20	0.158	193.07	323.57	534.2	21.4	24.96
30	0.244	188.79	304.57	498.2	19.2	25.95

*l^f^*: arithmetic average length; *l^f^_w_*: average length weighted in length; *l^f^_ww_*: average length double weighted.

**Table 3:** Data used to solve Kelly and Tyson modified equation by using Bowyer and Bader methodology.

Reinforcement weight content (%)	10	20	30
Reinforcement Volume fraction	0.077	0.158	0.244
Fiber modulus (GPa)	30.45	30.45	30.45
Elongation at break (%)	7.21	5.77	4.45
Strain level 1 analyzed (%)	2.38	1.90	1.48
Strain level 2 analyzed (%)	4.76	3.81	2.97
Composite stress at strain level 1 (MPa)	54.15	50.20	49.60
Composite stress at strain level 2 (MPa)	78.28	83.30	83.50
Matrix stress at break (MPa)	68.90	69.30	69.30
Matrix stress at strain level 1 (MPa)	46.46	38.11	30.60
Matrix stress at strain level 2 (MPa)	67.40	66.38	55.99

**Table 4:** Micromechanics tensile strength properties of PA6-based composites.

Reinforcement weight content (%)	10	20	30
Orientation factor χ_1_	0.30	0.30	0.28
Interfacial shear strength τ (MPa)	33.0	38.2	40.0
Critical length (μm)	354	309	283
Intrinsic tensile strength *σ_t_^f^* (MPa)	1051	1101	1178
